# Allele-specific activation, enzyme kinetics, and inhibitor sensitivities of EGFR exon 19 deletion mutations in lung cancer

**DOI:** 10.1073/pnas.2206588119

**Published:** 2022-07-22

**Authors:** Benjamin P. Brown, Yun-Kai Zhang, Soyeon Kim, Patrick Finneran, Yingjun Yan, Zhenfang Du, Jiyoon Kim, Abigail Leigh Hartzler, Michele L. LeNoue-Newton, Adam W. Smith, Jens Meiler, Christine M. Lovly

**Affiliations:** ^a^Chemical and Physical Biology Program, Vanderbilt University, Nashville, TN 37235;; ^b^Center for Structural Biology, Vanderbilt University, Nashville, TN 37232;; ^c^Department of Medicine, Division of Hematology and Oncology, Vanderbilt University Medical Center, Nashville, TN 37232;; ^d^Department of Chemistry, University of Akron, Akron, OH 44325;; ^e^Menten AI, San Francisco, CA 94111;; ^f^Vanderbilt-Ingram Cancer Center, Vanderbilt University Medical Center, Nashville, TN 37232;; ^g^Department of Chemistry, Vanderbilt University, Nashville, TN 37232;; ^h^Institute for Drug Discovery, Leipzig University Medical School, Leipzig, SAC 04103, Germany

**Keywords:** exon 19 deletion, EGFR, molecular dynamics, enzyme kinetics, lung cancer

## Abstract

Oncogenic mutations within the epidermal growth factor receptor (EGFR) are found in 15 to 30% of all non–small-cell lung carcinomas. The term exon 19 deletion (ex19del) is collectively used to refer to more than 20 distinct genomic alterations within exon 19 that comprise the most common EGFR mutation subtype in lung cancer. Despite this heterogeneity, clinical treatment decisions are made irrespective of which EGFR ex19del variant is present within the tumor, and there is a paucity of information regarding how individual ex19del variants influence protein structure and function. Herein, we identified allele-specific functional differences among ex19del variants attributable to recurring sequence and structure motifs. We built all-atom structural models of 60 ex19del variants identified in patients and combined molecular dynamics simulations with biochemical and biophysical experiments to analyze three ex19del mutations (E746_A750, E746_S752 > V, and L747_A750 > P). We demonstrate that sequence variation in ex19del alters oncogenic cell growth, dimerization propensity, enzyme kinetics, and tyrosine kinase inhibitor (TKI) sensitivity. We show that in contrast to E746_A750 and E746_S752 > V, the L747_A750 > P variant forms highly active ligand-independent dimers. Enzyme kinetic analysis and TKI inhibition experiments suggest that E746_S752 > V and L747_A750 > P display reduced TKI sensitivity due to decreased adenosine 5′-triphosphate *K*_m_. Through these analyses, we propose an expanded framework for interpreting ex19del variants and considerations for therapeutic intervention.

## Significance

Epidermal growth factor receptor (EGFR) mutations are detected in approximately 30% of all lung adenocarcinomas, and the most common EGFR mutation occurring in ∼50% of patients is termed “exon 19 deletion” (ex19del). Despite the existence of dozens of different genomic variants comprising what is generically referred to clinically as ex19del, clinicians currently do not distinguish between ex19del variants in considering treatment options, and differences between ex19del variants are largely unstudied in the broader scientific community. Herein, we describe functional differences between distinct EGFR ex19del variants attributable to the structural features of each variant. These findings suggest a possible explanation for observed differences in patient outcomes stratified by ex19del subtype and reinforce the need for allele-specific considerations in clinical treatment decision-making.

Oncogenic mutations within the epidermal growth factor receptor (EGFR) tyrosine kinase domain (KD) are detected in 15 to 30% of all cases of non–small-cell lung cancers (NSCLCs) (1, 2). The two most common EGFR KD mutations are a point mutation in exon 21, L858R, and a series of variants resulting in deletions within exon 19 (henceforward categorically referred to as ex19del mutations) (1, 2). More than a dozen genomic variants of ex19del have previously been identified (3, 4). Historically, ex19del mutations have not been differentiated in the clinic, and despite the known heterogeneity within this cohort of *EGFR*-mutant lung cancer, variant-specific differences in ex19del have not been widely considered.

This is in stark contrast to the less frequently occurring EGFR exon 20 insertion (ex20ins) mutations. Several reports have described in detail the heterogeneity that different ex20ins variants display in terms of enzymatic activity and sensitivity to existing Food and Drug Administration–approved tyrosine kinase inhibitors (TKIs) (5–9). At the structural level, molecular dynamics (MD) simulations suggest that ex20ins mutants can lower the free energy barrier associated with adopting the KD active conformation in an allele-specific manner (10). There are multiple ongoing drug development efforts aimed at designing TKIs to treat tumors harboring ex20ins variants in an allele-specific way (11–13). Preliminary assessment of an ongoing clinical trial (NCT03974022) suggests that this approach may be efficacious in ex20ins (14). The case has been made that we must evaluate drug efficacy on a per-mutant basis for ex20ins while ironically grouping all ex19del variants together (15). However, several retrospective studies have now suggested that there are differences in patient outcomes between ex19del patient populations (3, 4, 16–19). Emerging evidence suggests that structural classification of EGFR mutants can improve retrospective prediction of drug sensitivities (20). The lack of allele-specific resolution of ex19del variants in clinical practice may impede our ability to provide optimal therapeutic strategies for patients with NSCLC and other cancers.

It is also noteworthy that investigations into ex19del often use the verbiage “exon 19 deletion” to refer to different allele variants, making it more challenging to functionally characterize them and develop appropriate therapeutic strategies. For example, the mechanism of activation of ex19del has been reported to be both ligand independent (21–24) and ligand dependent (25–27), and it is unclear to what extent the discrepancy is a result of the use of different experimental methodologies or different ex19del variants evaluated in previous studies. We also previously found that the development of osimertinib resistance to the G724S mutant is dependent on the specific ex19del variant (28), suggesting that ex19del structural differences can have therapeutic implications. Thus, to maximize the efficacy of targeted therapies, we need to refine our understanding of oncogenic variants at the atomic level.

In this study, we tested the hypothesis that sequence variation between EGFR oncogenic ex19del mutations can lead to allele-specific activation and TKI sensitivity. We probed the American Association for Cancer Research (AACR) Genomics Evidence Neoplasia Information Exchange (GENIE) database (29) and identified 60 unique ex19dels and built structural models of each variant. Next, we selected three of the most common variants predicted to be structurally distinct for detailed computational, biophysical, and biochemical evaluation: E746_A750, E746_S752 > V, and L747_A750 > P. Altogether, our results demonstrate that ex19dels are a functionally heterogeneous population with potentially unique considerations for optimal therapeutic targeting.

## Results

### Ex19del Sequence Variants Cluster by Chemical Conservation and Thus Function.

**Fig. 1.**
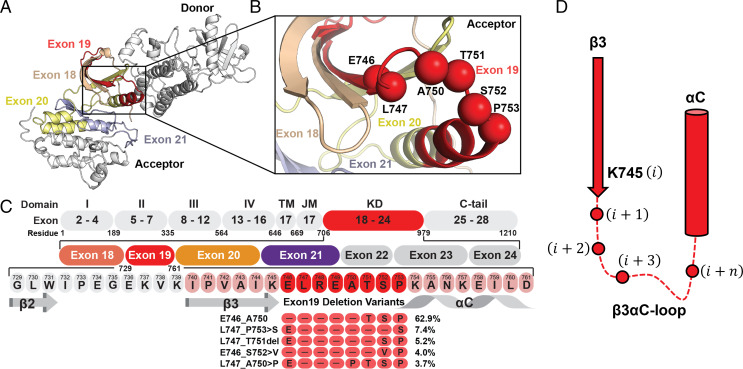
Frequently occurring mutations in the EGFR β3-αC motif. (*A*) Schematic representation of the active EGFR-WT asymmetric dimer. Oncogenic and TKI resistance mutations have been reported in exons 18 (wheat), 19 (red), 20 (yellow), and 21 (blue). (*B*) The majority of deletion mutations begin at residues E746, L747, or T751. Deletion mutants frequently terminate with or without an insertion at position A750, T751, S752, or P753. Spheres indicate the residue Cα. (*C*) Multiple sequence alignment of the β3-αC motif between EGFR-WT and ex19del variants with >2% frequency. (*D*) Residues at the β3-αC interface can be referenced with respect to their index after the conserved K745 residue in the majority of mutants.

We first investigated the sequence heterogeneity of ex19del variants by probing the AACR GENIE database (29). We identified 60 variants and mapped these variants to the EGFR KD (Fig. 1 and *SI Appendix*, Table S1). Structurally, exon 19 corresponds to the β3 sheet, β3-αC loop, and N-terminal half of theαC helix (Fig. 1*A*). All residues are numbered with respect to wild type (WT) in the immature form (e.g., we reference L858R instead of L834R). We identified mutants ranging in size from a single-residue deletion to a net eight-residue deletion (*SI Appendix*, Table S1). The starting and stopping points for the deletions predominantly occurred at residues E746, L747, A750, T751, S752, and P753, such that the length of the β3-αC loop is the primary subject of sequence variation when compared to the β3 or αC regions (Fig. 1*B* and *SI Appendix*, Table S1). The predominant mutations are E746_A750 (62.9%), L747_P753 > S (7.4%), L747_T751 (5.2%), E746_S752 > V (4.0%), and L747_A750 > P (3.7%) (Fig. 1*C* and *SI Appendix*, Table S1).

The breadth of variants is substantial, ranging from deletions that occur entirely in β3 (K739_I744 > N) to those occurring almost entirely in αC (e.g., P753_I759). To help characterize the mutations, we first built structural models of all variants utilizing the Rosetta comparative modeling approach coupled with Gaussian accelerated MD (GaMD) (30) (see *Materials and Methods*). Our models suggested several recurring structural features of ex19del. First, the most common ex19del variants, including E746_A750, L747_P753 > S, and L747_T751 (Fig. 1*C*), replace L747 at the β3-αC interface with a serine and simultaneously remove at least one full turn from the N terminus of the αC helix (*SI Appendix*, Fig. S1*A*). Second, mutants with net deletions of size three, such as L747_A750 > P and E746_T751 > APS, frequently converge on the same β3-αC loop conformation, characterized by a β3-αC tight turn with proline in the second position (*SI Appendix*, Fig. S1*B*–*D*). Third, we observed that several mutants project polar residues into the adenosine 5′-triphosphate (ATP)–binding pocket in the vicinity of the canonical K745 – E762 salt bridge, such as L747_S752 > Q and E746_S752 > V (cis-trans proline-dependent).

To evaluate potential functional differences between mutants, we selected three isoforms that are prevalent in patients based on our AACR GENIE analysis (Fig. 1*C*) and that cover the breadth of features described above: E746_A750 (62.9%), E746_S752 > V (4.0%), and L747_A750 > P (3.7%). For clarity, we periodically reference residues by their position relative to K745 (Fig. 1*D*).

### Ex19del Variants Adopt Unique β3-αC Conformations with Different Energetic Barriers to Activation.

We began with the hypothesis that ex19dels can display allele-specific differences in their propensity to adopt the active conformation. Wild-type EGFR (WT) is activated when ligand binds the extracellular domain (ECD) to promote intermolecular dimerization and further oligomerization (31–33). Intracellularly, these conformational changes result in asymmetric dimerization between two KDs where the “receiver” KD is stabilized in an active conformation by the “donor” KD (34). Previous investigations have shown that oncogenic variants in the KD often stabilize the αC- helix by suppressing intrinsic disorder (35), leading to enhanced dimerization where the mutant KD behaves as a “super acceptor” (36).

We performed six independent conventional molecular dynamics (cMD) simulations of 4.0 to 6.0 μs for each mutant and state (WT, E746_A750, E746_S752 > V, and L747_A750 > P in active and inactive states), such that three simulations were initiated from each state (120.0 μs total). Consistent with previous reports (37), the αC helix of WT readily departed from the active conformation to adopt an unstructured intermediate state, and 1/3 active state simulations transitioned completely to the Src-like inactive conformation (αC helix out, A-loop in, DFG in) (*SI Appendix*, Fig. S2*A* and Movie S1).

In comparison, each of the ex19del variants was stabilized in the active state (αC helix in, A-loop out, DFG in; *SI Appendix*, Fig. S2*B*–*D*). The tight turn predicted in the Rosetta/GaMD model of L747_A750 > P is restricted in its motion, preventing inactivation (*SI Appendix*, Fig. S2*D*). Unfortunately, no transitions were observed from the inactive to the active state or vice versa in any of the ex19del cMD simulations. Therefore, we combined steered MD (SMD) with umbrella sampling (UMD) simulations to map the conformational free energy landscape (FEL) of the transition.

**Fig. 2.**
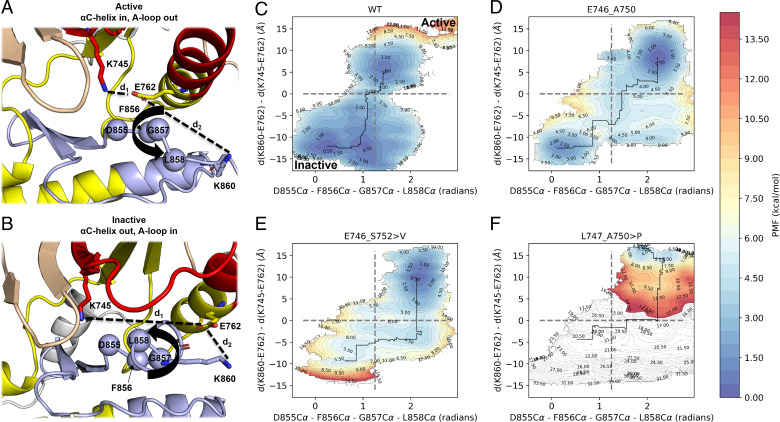
Conformational free energy landscapes of ex19del variants from UMD simulations. (*A* and *B*) CVs describe the (*A*) active and (*B*) inactive states as the pseudodihedral angle formed by the alpha carbon atoms of residues D855, F856, G857, and L858 (x-axis; bold arrowhead line) as well as the difference in distance between the capping side chain atoms of E762 and K745 (d1) and E762 and K860 (d2) (*y* axis; black dashed lines). (*C*–*F*) Conformational free energies are shown for (*C*) WT, (*D*) E746_A750, (*E*) E746_S752 > V, and (*F*) L747_A750 > P. Gray dashed lines separate the inactive (bottom left quadrant) from the active (top right quadrant) states. Plots are contoured at 0.5 kcal/mol and colored within the range 0 (blue) and 15 (red) kcal/mol. Contours above 15 kcal/mol are colored white. PMF, potential of mean force.

Following a procedure similar to that previously employed for ex20ins variants (10), we defined our UMD collective variables (CVs) along two dimensions: 1) Activation state of the αC helix as defined by the difference in distance between K860 – E762 and K745 – E762 and 2) activation state of the A-loop as defined by the dihedral angle formed by the Cα atoms of D855 – F856 – G857 – L858 (Fig. 2*A* and *B*).

Using these two CVs, we measured the free energy difference between the active and inactive states of WT and found it to be ∼1.0 kcal/mol in favor of the inactive state (Fig. 2*C*), in good agreement with prior estimates (10). In contrast to WT and the previously reported ex20ins mutations (10), all three ex19del variants favored the active state (Fig. 2*D*–*F*). E746_A750 and E746_S752 > V favored the active state by ∼1.0 kcal/mol and 4.5 kcal/mol, respectively (Fig. 2*D* and *E*). We also performed SMD+UMD simulations on the other two most commonly occurring ex19dels, L747_P753 > S and L747_T751. L747_T751 displayed an activation profile similar to that of E746_S752 > V, while L747_P753 > S may be more comparable to several ex20ins variants (10) (*SI Appendix*, Fig. S3).

Interestingly, L747_A750 > P appears to be trapped in the active state, with prohibitively large free energy barriers to the inactive state (Fig. 2*F*). We considered that this may be a result of the proline substitution at position 747. We tested this hypothesis by building models for the oncogenic missense variant L747P (38) and performing SMD+UMD simulations. L747P induced an ordered tight turn in the β3-αC loop (*SI Appendix*, Fig. S1*C*), stabilizing the active state over the inactive state by ∼1.0 kcal/mol (*SI Appendix*, Fig. S3*C*), but not by as large a margin as L747_A750 > P. The substantially larger barrier to inactivation in L747_A750 > P may result from the proline in its β3-αC tight turn coupled with the net three-residue deletion (*SI Appendix*, Fig. S1*D*). Altogether, our results suggest that ex19del variants adopt unique conformations near the receiver KD interface that translate into potentially substantial differences in activation propensity.

### L747_A750 > P, but not E746_A750 or E746_S752 > V, Dimerizes in a Ligand-Independent Manner.

Previous studies have suggested that KD mutants may promote ligand-dependent “inside-out” dimerization (39). Based on our simulation results, we hypothesized that the L747_A750 > P variant forms dimers in the absence of ligand stimulation because it is trapped in a receiver kinase active state. To test our hypothesis, we measured the homo-interaction stoichiometry of each variant in the presence and absence of epidermal growth factor (EGF) ligand using two-color pulsed interleaved excitation fluorescence cross-correlation spectroscopy (PIE-FCCS) (33, 40). Live-cell PIE-FCCS measurements and analysis were completed on single cells expressing individual ex19del variants with WT data recorded as a negative control for each experiment (see *Materials and Methods*).

**Fig. 3.**
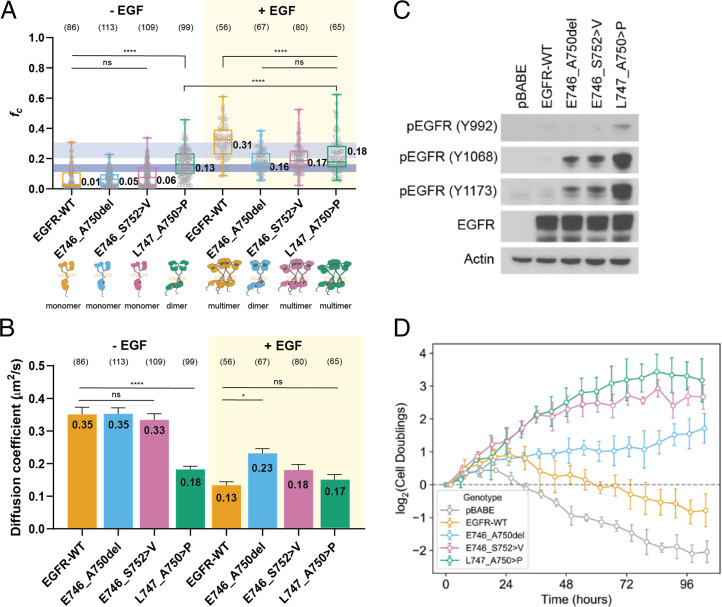
Ex19del variants display allele-specific differences in dimerization and oncogenic growth. (*A*) Cross-correlation values of transfected EGFR variants with (+) or without (-) ligand (EGF) stimulation. The dark and light blue boxes indicate the *ƒ_c_* value regions for dimers and multimers, respectively. Data are presented as a box and whiskers plot, the whiskers show the maximum and the minimum; the box shows 25th–75th percentile; and the line in the box is the median value. The median values are reported next to the boxplot. Each gray dot represents the averaged acquisition (10 s, 6 acquisitions) per area per cell. All data points are shown. One-Way ANOVA test with Bonferroni post-hoc test was performed to obtain the adjusted P values. ****P <0.0001; *P <0.05; ns, not significant. (*B*) Diffusion coefficient values of EGFR variants with (+) or without (-) ligand (EGF) stimulation. Data are presented as mean values ± SD. One-Way ANOVA test with Bonferroni post-hoc test was performed to obtain the adjusted P values. ****P <0.0001; *P <0.05; ns, not significant. The light orange box indicates EGF-stimulated groups. (*C*) Ba/F3 cells were stably transfected with different EGFR ex19del variants, WT, or empty vector. Cellular lysates were probed with the indicated antibodies to measure phosphorylation. (*D*) Rate of IL-3–independent growth of Ba/F3 cells stably transfected with different ex19del variants, WT, or empty vector. ns, not significant.

First, we performed PIE-FCCS experiments in the absence of EGF ligand. Samples were serum starved for 24 h to ensure no residual ligand-dependent effects. Protein expression levels were measured experimentally and ranged from 158 to 2,381 receptors/µm^2^, which is consistent with the normal physiological expression of EGFR (41). As expected, WT had a median cross-correlation (ƒ_c_) value near zero (ƒ_c_ = 0.01), indicating that it exists predominantly as a monomer. Our results also suggest that E746_A750 and E746_S752 > V are predominantly monomeric in the absence of ligand (ƒ_c_ = 0.05 and 0.06, respectively). In contrast, L747_A750 > P displayed significantly higher median cross correlation (ƒ_c_ = 0.13) (Fig. 3*A*). Consistent with the cross-correlation values, the diffusion coefficients of enhanced green fluorescent protein–tagged WT (0.35 μm^2^/s), E746_A750 (0.35 μm^2^/s), and E746_S752 > V (0.33 μm^2^/s) were significantly higher than that of L747_A750 > P (0.18 μm^2^/s) (Fig. 3*B*). The increased median cross correlation and decreased diffusion coefficient of L747_A750 > P relative to WT is indicative of dimer formation in the absence of ligand stimulation.

Next, we performed PIE-FCCS experiments in the presence of EGF ligand to evaluate whether or not ex19del variants differ in their response to extracellular stimulation. A recent study demonstrated that KD mutations can directly change the conformational preferences of the ECD, potentially modulating signaling responses to ligand (42). Here, we observed that WT forms multimers upon stimulation with EGF, consistent with prior studies (ƒ_c_ = 0.31; *D* = 0.13 μm^2^/s) (32, 33, 40, 43). EGF stimulation caused E746_A750 (ƒ_c_ = 0.16; *D* = 0.23 μm^2^/s), E746_S752 > V (ƒ_c_ = 0.17; *D* = 0.18 μm^2^/s), and L747_A750 > P (ƒ_c_ = 0.18; *D* = 0.17 μm^2^/s) to form a mixture of dimers and multimers (Fig. 3*A* and *B*). The fact that each of the mutants showed lower cross correlation and faster diffusion compared to WT suggests that the ex19del mutations may have an inhibitory effect on the formation of ligand-dependent multimeric assemblies.

### E746_S752 > V and L747_A750 > P Display Enhanced Oncogenic Activation Relative to E746_A750.

The strong energetic preference of L747_A750 > P to adopt the active conformation (Fig. 2*F*) and corresponding propensity to form ligand-independent dimers (Fig. 3*A* and *B*) led us to hypothesize that L747_A750 > P would display enhanced oncogenic growth compared with other ex19del variants in vitro. To test our hypothesis, we generated expression vectors containing empty vector, WT, E746_A750, E746_S752 > V, or L747_A750 > P and introduced these into murine lymphoid Ba/F3 cells (44). After selection of stable expression in puromycin, the cells were collected, lysed, and blotted for EGFR autophosphorylation (pEGFR). Our results confirmed that all three ex19del variants exhibit strong pEGFR compared to WT. In support of our hypothesis, we observed that L747_A750 > P displays substantially higher levels of pEGFR compared with either E746_A750 or E746_S752 > V (Fig. 3*C*).

To further investigate ex19del variant differences in interleukin (IL)-3–independent oncogenic growth in Ba/F3 cells, we depleted IL-3 from the growth medium to monitor changes in cell counts over time (Fig. 3*D*). As expected, the Ba/F3 cells expressing either vector or WT EGFR died shortly upon withdrawal of exogenous IL-3, while cells expressing EGFR ex19del variants survived and proliferated. Cells expressing either E746_S752 > V or L747_A750 > P proliferated at a higher rate compared with cells expressing E746_A750 (Fig. 3*D*). Despite not undergoing ligand-independent dimerization as did L747_A750 > P in PIE-FCCS experiments, cells expressing E746_S752 > V displayed statistically similar growth rates compared with L747_A750 > P. Collectively with our MD simulations, our results suggest that ex19del variants differentially promote growth and enzymatic activity and that this could at least in part be due to differences in their activation FEL.

### E746_S752 > V and L747_A750 > P Are Less Sensitive to TKI Treatment Than E746_A750.

We considered the possibility that differences may exist between ex19del variant TKI sensitivities, which may explain differences in outcomes between patients with specific ex19dels (4, 19). We previously found that some ex19del variants, in particular E746_S752 > V, are especially likely to develop G724S-mediated resistance in response to osimertinib, while L858R and other ex19del variants are not (28, 45). Recently, it was further suggested that L747_A750 > P has reduced sensitivity to erlotinib and osimertinib relative to E746_A750 in functional assays due to steric effects (46). Thus, we sought to evaluate the relative TKI sensitivity of E746_A750 in comparison to that of E746_S752 > V and L747_A750 > P.

**Fig. 4.**
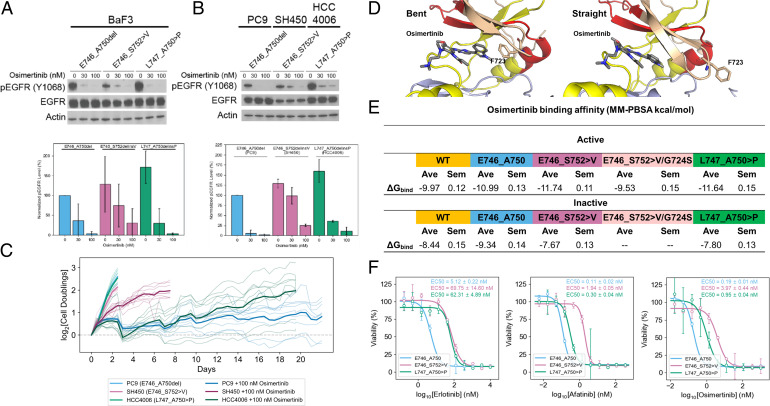
Allele-specific differences in ex19del TKI sensitivity may not be due to differences in TKI binding affinity. (*A*) Ba/F3 cells were stably transfected with different EGFR ex19del variants and treated with increasing concentrations (0, 30, or 100 nM) of osimertinib. Cellular lysates were probed with the indicated antibodies to measure phosphorylation. Quantifications are represented as the average pEGFR normalized to E746_A750 in the absence of osimertinib ± SD across three independent biological replicates. (*B*) Lung adenocarcinoma cell lines expressing E746_A750 (PC9), E746_S752 > V (SH450), or L747_A750 > P (HCC4006) were treated with increasing concentrations (0, 30, or 100 nM) of osimertinib. Cellular lysates were probed with the indicated antibodies to measure phosphorylation. Quantifications are represented as the average pEGFR normalized to E746_A750 in the absence of osimertinib ± SD across three independent biological replicates. (*C*) Time-dependent growth of lung adenocarcinoma cell lines expressing E746_A750 (PC9), E746_S752 > V (SH450), or L747_A750 > P (HCC4006) treated with either 100 nM osimertinib or buffer. Each condition was performed five times (thin lines) and averaged (bold lines). (*D*) Structural models of EGFR in complex with osimertinib in either the bent (F723 facing osimertinib in the ATP-binding pocket) or straight (F723 projecting away from the ATP-binding pocket) conformations. (*E*) Osimertinib-binding affinities for each ex19del variant, WT, and the double mutant E746_S752 > V/G724S from simulations starting in the active and inactive states. Binding energies are computed as the average (Ave) and SEM (Sem) MM-PBSA energies of 1,000 randomly selected frames from the equilibrated ensembles. For each EGFR variant, six simulations of 2.0 μs each were performed such that there were three each from the active and inactive states (except E746_S752 > V/G724S, for which no inactive state simulations were performed). (*F*) TKI sensitivities of Ba/F3 cells expressing EGFR mutants. Cell viability assays performed in Ba/F3 cells stably expressing E746_A750 (blue), E746_S752 > V (pink), or L747_A750 > P (green) with erlotinib (first panel), afatinib (second panel), or osimertinib (third panel). Three biological replicates performed for each mutant/TKI combination. Representative plots displayed. EC50 values are reported as mean ± SEM over the three independent replicates. EC50, effective concentration, 50%.

We first treated Ba/F3 cells expressing E746_A750, E746_S752 > V, or L747_A750 > P with either 30 or 100 nM osimertinib. We observed that autophosphorylation was markedly reduced in both E746_A750 and L747_A750 > P, but not in E746_S752 > V (Fig. 4*A*). Subsequently, we performed the same experiment in well-established lung adenocarcinoma cell lines expressing E746_A750 (PC9), E746_S752 > V (SH450), or L747_A750 > P (HCC4006). Again, we observed that E746_S752 > V was less sensitive to osimertinib than E746_A750 or L747_A750 > P (Fig. 4*B*). To model the clinical exposure of EGFR TKIs in lung adenocarcinoma, we performed long-term treatments of osimertinib in these cell lines at a clinically relevant dose (100 nM) (47) with periodic medium/TKI refreshment (Fig. 4*C*). The untreated PC9, SH450, and HCC4006 cells underwent exponential growth and quickly reached confluence within 3 d. The growths of PC9 and HCC4006 cells were inhibited effectively by osimertinib treatment, and the cells initially stopped growing. In particular, the proliferation of PC9 cells was successfully inhibited by osimertinib for more than 3 wk. We observed that the HCC4006 cells gradually adapted to the treatment and proliferated to confluence in 20 d. Most notably, however, osimertinib only partially inhibited the proliferation of SH450 cells and, after an incomplete response, continued growing, reaching confluence within a week. Thus, consistent with our Western blots, we found that E746_S752 > V was least responsive to osimertinib, followed by L747_A750 > P, while E746_A750 was completely inhibited (Fig. 4*C*).

Based on the in vitro data, we hypothesized that E746_S752 > V has a lower osimertinib binding affinity than E746_A750 and L747_A750 > P. To test this hypothesis, we performed MD simulations of each of the ex19del variants in complex with osimertinib. We performed three independent MD simulations of 2.0 μs each for each EGFR variant (WT, E746_A750, E746_S752 > V, E746_S752 > V/G724S, or L747_A750 > P) bound to osimertinib starting from either the active or inactive conformation (sans inactive E746_S752 > V/G724S; 60.0-μs aggregate simulation time). As expected based on the available crystallographic evidence (48), osimertinib binding energies suggested tighter binding in the active state than the inactive state in all cases. Both E746_A750 and L747_A750 > P were estimated to have a better osimertinib binding free energy than WT (Fig. 4*E*). Contrary to our hypothesis, E746_S752 > V was not predicted to bind osimertinib with a lower affinity than E746_A750. In contrast to previous studies (46), L747_A750 > P failed to show a reduced osimertinib binding free energy (Fig. 4*E*).

To better understand our simulation results, we quantitatively evaluated the inhibitory efficacy of three generations of EGFR TKIs (erlotinib, afatinib, and osimertinib) by measuring cell viabilities of isogenic Ba/F3 cells stably transfected with either E746_A750, E746_S752 > V, or L747_A750 > P in the presence of each TKI separately. We observed that L747_A750 > P and E746_S752 > V were both at least 10x less sensitive to TKI than E746_A750 (Fig. 4*F*). We corroborated these results by measuring cell viabilities of lung adenocarcinoma cell lines expressing different ex19del variants. Here, we also observed that SH450 (E746_S752 > V) or HCC4006 (L747_A750 > P) were at least 10x less sensitive to erlotinib than PC9 (E746_A750). SH450 was also greater than 10x less sensitive to afatinib and osimertinib as compared to PC9 or HCC4006 (*SI Appendix*, Fig. S5). L747_A750 > P displayed a similar response to afatinib as E746_A750. Our results suggest that E746_S752 > V and L747_A750 > P are intrinsically less sensitive to ATP-competitive TKIs in vitro. E746_A750 displayed the most TKI sensitivity among the three ex19dels.

### Differences in ATP Binding May Modulate TKI Sensitivity across ex19del Variants.

Our in vitro data suggest that E746_S752 > V and L747_A750 > P display reduced sensitivity to standard first-, second-, and third-generation TKIs. Simultaneously, our MD simulations estimate that E746_S752 > V and L747_A750 > P reversibly bind osimertinib at least as well as E746_A750, if not more tightly. Thus, we hypothesized that the reduced sensitivity of E746_S752 > V or L747_A750 > P to ATP-competitive inhibitors is the result of higher ATP-binding affinities in these receptors than in other EGFR oncogenic variants, thereby reducing the relative binding affinity of TKI to ATP.

**Fig. 5.**
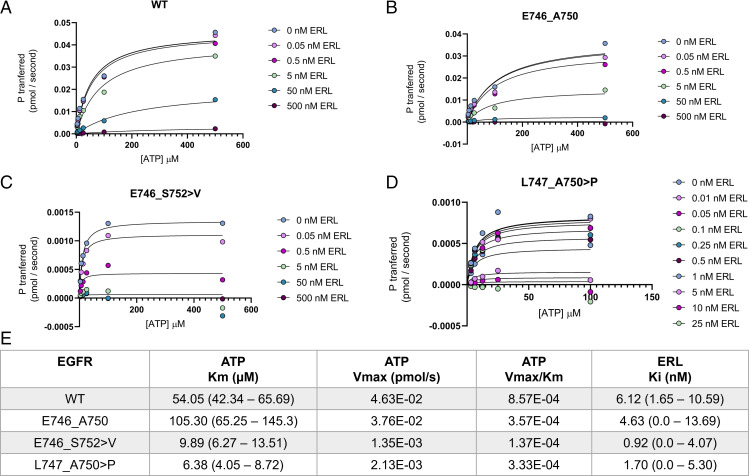
Allele-specific differences in enzyme kinetics contributes to variability in TKI sensitivity. (*A–D*) Michaelis-Menten steady-state kinetics of EGFR (*A*) WT, (*B*) E746_A750, (*C*) E746_S752 > V, and (*D*) L747_A750 > P at varying concentrations of the ATP-competitive noncovalent TKI erlotinib (ERL) and substrate ATP at 0.2 µg/µL of peptide substrate Poly (4:1 Glu, Tyr) as determined by the ADP-Glo assay. Rate is expressed as phosphate transferred in picomole per second. (*E*) Enzyme kinetic parameters and erlotinib-binding affinity for EGFR WT and ex19del variants. Data from (*A*–*D*) were fit with least-squares to a mixed model of inhibition in GraphPad Prism 9.3.1 and are reported as best-fit values. The value ranges in parentheses indicate 95% confidence intervals. Vmax, the maximum rate in a Michaelis-Menten system.

To test this hypothesis, we estimated the apparent ATP *K*_m_ and erlotinib *K*_i_ for WT, E746_A750, E746_S752 > V, and L747_A750 > P and for the additional uncommon variant L747_E749. We chose erlotinib for the TKI-binding affinity analysis to enable comparison of the effects of ATP *K*_m_ on noncovalent TKI interactions. Our results suggest that there are substantial differences in ATP kinetics between EGFR variants (Fig. 5 and *SI Appendix*, Fig. S6).

Specifically, E746_A750 and L747_E749 displayed ATP *K*_m_ values of ∼100 µM (Fig. 5*B*, *D*, and *E* and *SI Appendix*, Fig. S6*A* and *B*). In contrast, E746_S752 > V and L747_A750 > P displayed ATP *K*_m_ < 10 µM (Fig. 5*C*–*E*). The rates of phosphate transfer in both E746_S752 > V and L747_A750 > P were ∼16 to 25x lower than that of E746_A750, but the reduced *K*_m_ values resulted in comparable catalytic efficiencies (Fig. 5*E*). Interestingly, phosphate transfer in both E746_S752 > V and L747_A750 > P was more robustly inhibited than in either WT or E746_A750 (Fig. 5*E*). These results are consistent with our MD simulations (Fig. 4*E*) as well as with the reduced sensitivity of E746_S752 > V and L747_A750 > P in vitro. Overall, these data suggest that differences in ATP kinetics may differentially sensitize ex19del variants to TKI.

Our simulations create structural context and suggest several hypotheses for these differences. First, ex19del variants make distinct hydrogen bonding interactions at the β3-αC interface (*SI Appendix*, Fig. S7*A*–*D*). E746_A750 places S752 at the β3-αC i+2 position (Fig. 1*D*) such that the side chain donates a H-bond to the F723 backbone and is simultaneously stabilized as a H-bond acceptor from the K754 backbone (*SI Appendix*, Fig. S7*B*). Neither E746_S752 > V nor L747_A750 > P, both of which place a proline at i+2, can make this H-bond (*SI Appendix*, Fig. S7*C* and *D*). Quantitation of apo-state H-bonding supports this observation, suggesting that the glycine-rich loop is more tightly coupled to the β3-αC loop in E746_A750 (*SI Appendix*, Fig. S7*E*). These data, together with previous crystallographic (49) and kinetic (50) studies of EGFR L858R, suggest generally that tight coupling of the β3-αC loop to the glycine-rich loop in αC helix–stabilizing oncogenic mutants may lead to reduced ATP-binding affinity.

### New Therapeutic Strategies May Be Required to Maximally Inhibit E746_S752 > V–Mediated Disease.

**Fig. 6.**
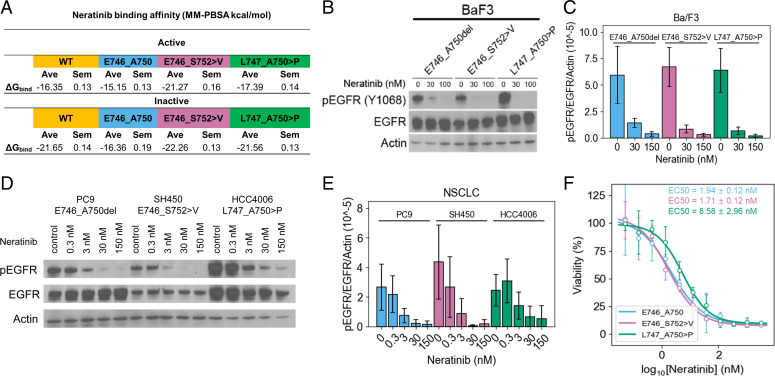
Neratinib effectively inhibits E746_S752 > V. (*A*) Neratinib-binding affinities for each ex19del variant and WT from simulations starting in the active and inactive states. Binding energies are computed as the average (Ave) and SEM (Sem) MM-PBSA energies of 1,000 randomly selected frames from the equilibrated ensembles. For each EGFR variant, six simulations of 2.0 μs each were performed such that there were three each from the active and inactive states. (*B*) Ba/F3 cells were stably transfected with different EGFR ex19del variants and treated with increasing concentrations (0, 30, or 100 nM) of neratinib. Cellular lysates were probed with the indicated antibodies to measure phosphorylation. (*C*) Quantification of Ba/F3 neratinib inhibition. Western blots are represented as the average pEGFR/EGFR normalized to actin ± SD across three independent biological replicates. (*D*) Lung adenocarcinoma cell lines expressing E746_A750 (PC9), E746_S752 > V (SH450), or L747_A750 > P (HCC4006) were treated with increasing concentrations (0, 30, or 100 nM) of neratinib. Cellular lysates were probed with the indicated antibodies to measure phosphorylation. (*E*) Quantification of lung adenocarcinoma cell line neratinib inhibition. Western blots are represented as the average pEGFR/EGFR normalized to actin ± SD across three independent biological replicates. (*F*) TKI sensitivities of Ba/F3 cells expressing EGFR mutants. Cell viability assays performed in Ba/F3 cells stably expressing E746_A750 (blue), E746_S752 > V (pink), or L747_A750 > P (green) with neratinib. Three biological replicates performed for each mutant/TKI combination. Representative plots displayed. EC50 values are reported as mean ± SEM over the three independent replicates. EC50, effective concentration, 50%.

We previously identified the TKI neratinib as a potential therapeutic agent for certain forms of HER2/HER3-mutant cancers in which pan-TKI resistance seems to be associated with enhanced ATP-binding affinity (51). Employing the same strategy for neratinib as we did for osimertinib, we performed MD simulations and subsequent Molecular Mechanics Poisson-Boltzmann Surface Area (MM-PBSA)-binding free energy estimates of ex19dels complexed with neratinib. Our simulations suggest that all of the tested ex19dels reversibly bind neratinib better than osimertinib, but that E746_S752 > V has a better neratinib-binding energy than E746_A750 or L747_A750 > P (Fig. 6*A*). Evaluation of neratinib function inhibition in Ba/F3 cells stably transfected with E746_A750, E746_S752 > V, or L747_A750 > P demonstrated a complete ablation of pEGFR in E746_S752 > V and L747_A750 > P at 30 nM. Phosphorylation was largely reduced in E746_A750 at 30 nM and completely ablated at 150 nM (clinical-relevant dose; Fig. 6*B* and *C*). We also observed that neratinib effectively reduced pEGFR in Ba/F3 cells and lung adenocarcinoma cell lines expressing E746_A750, E746_S752 > V, or L747_A750 > P (Fig. 6*D*–*F* and *SI Appendix*, Fig. S8).

## Discussion

Considerable effort has been invested over the last decade to define the molecular mechanisms of oncogenesis and acquired drug resistance in the most commonly occurring *EGFR* mutations, specifically L858R and ex19del (26, 27, 34–36, 50). These efforts have resulted in the development of more effective targeted therapies, including today’s first-line therapy for *EGFR*-mutant NSCLC, osimertinib (52). Despite next-generation sequencing having identified heterogeneity in the various distinct ex19del variants, the allele-specific mechanisms have not been extensively evaluated. The potential reduced likelihood of noncanonical ex19del variants developing T790M or C797S in response to first- or third-generation TKI, respectively (16, 53), may be because a number of these variants have reduced TKI sensitivity in the setting of higher ATP-binding affinity. Indeed, both our group (28) and others (45) found that the G724S resistance mutation occurred preferentially to C797S in E746_S752 > V and related noncanonical variants in response to osimertinib. However, at present, there has not been a systematic evaluation of patient responses to different TKIs based on the specific ex19del variant present in tumor. Thus, it is imperative that we investigate individual ex19del variants preclinically to ultimately help guide clinicians in therapeutic decision-making.

Here, we performed computational, biophysical, and biochemical analyses on a diverse subset of the most frequently occurring ex19del variants: E746_A750, E746_S752 > V, and L747_A750 > P. Our data show clear differences in the activation profiles, enzyme kinetics, and TKI sensitivities of these ex19del variants with potential structural correlates. Specifically, our data suggest that the ligand dependency of receptor activation differs between ex19dels. The L747_A750 > P mutant displayed robust αC helix stabilization from a proline-locked tight turn in MD simulations that translated to ligand-independent dimerization and increased in vitro activity in experiments. We also observed that E746_S752 > V and L747_A750 > P were less sensitive to inhibition by TKI than E746_A750. We were unable to attribute this effect to binding affinity based on MD simulations of osimertinib or ADP-Glo inhibition assays for erlotinib. Instead, our data suggest a role for variable ATP K_M_ as a potential mediator of these differences in TKI sensitivity. It was previously observed that some oncogenic EGFR mutations can modulate ATP binding and TKI sensitivity (26, 27, 50, 54).

**Fig. 7.**
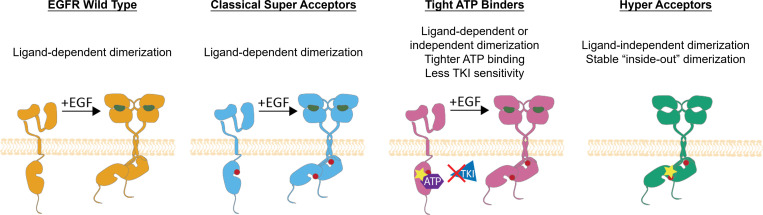
Model of ex19del allele-specific functional differences and strategy for inhibition. Discretized classification scheme for EGFR ex19del variants: nononcogenic with ligand-dependent activation (orange; WT); oncogenic super acceptor with ligand-dependent activation (blue; E746_A750, E746_S752 > V); tight ATP binder (pink; E746_S752 > V, L747_A750 > P); oncogenic hyper acceptor with ligand-independent activation (green; L747_A750 > P).

Collectively, our data demonstrate that ex19dels are a heterogeneous group of oncogenic variants. EGFR WT is a monomer in the absence of ligand and is stimulated by extracellular EGF to form dimers and multimers/oligomers (Fig. 7, orange). The most frequently occurring ex19del oncogenic mutants, such as E746_A750, increase the propensity for dimerization by stabilizing the acceptor KD (Fig. 7, blue). These “classical super acceptors” (35, 36) are ligand-dependent and have lower ATP-binding affinity (26), increasing their sensitivity to TKIs with lower reversible binding affinity, such as osimertinib (55). Our simulations and TKI sensitivity data suggest that a subset of ex19del variants, such as E746_S752 > V and L747_A750 > P, are “tight ATP binders” (Fig. 7, pink). These are characterized by ATP-binding affinities higher than that of classical super acceptors, making them more resistant to ATP-competitive TKIs, reminiscent of T790M-comutant EGFR (50) (Fig. 7). Finally, another subset of ex19dels, such as L747_A750 > P, are characterized by enhanced dimerization propensities greater than that of super acceptors. These “hyper acceptors” display increased functional activation and exist as ligand-independent dimers (Fig. 7, green). The ligand-independent activity of hyper acceptors suggests that some oncogenic variants may be activated via inside-out dimerization.

Based on our proposed model, L747_A750 > P is both a hyper acceptor and a tight ATP binder, while E746_S752 > V is a classical super acceptor and a tight ATP binder. E746_A750 is strictly a classical super acceptor. We suggest that ex19del variants likely exist along a spectrum of dimerization propensities and ATP affinities and anticipate that additional functional characterization of ex19del variants along these axes will allow more personalized treatment of ex19del NSCLC patients. To facilitate future structural comparisons of ex19del variants, we have made our computational structural models of these variants available (see *Data Availability*).

Generally, our data lead us to suggest that treatment of ex19del variants may require unique consideration of the variant’s functional properties. For example, we speculate that mutations with enhanced ligand-independent dimerization would be less amenable to EGF-blocking antibody/TKI combination therapies than classical super acceptor–like variants. We also suggest that for ex19dels with high ATP-binding affinities, the use of covalent TKIs with higher reversible binding affinities may be necessary to overcome reduced TKI sensitivity, such as neratinib or mobocertinib. Alternatively, because increasing the reversible binding affinity on covalent inhibitors can reduce mutant selectivity and cause undesirable side effects, recognition of tight ATP-binding ex19dels may motivate the design of PROTAC or allosteric inhibitors.

This study is not a comprehensive guide to EGFR ex19del variants. We hope that subsequent work expands upon this study to better characterize uncommon ex19dels. While in silico modeling can provide useful insight to generate hypotheses, it can be limited by factors such as the quality of the predicted structures, the short simulation timescales available to us, the start- and end-state dependency of UMD simulations, and the simplification of the system from transmembrane dimers/multimers to monomeric intracellular KDs. Similarly, in vitro data in the absence of structural characterization and dynamical insight can make it challenging to generalize findings and perform rational drug design. We anticipate that continued characterization of ex19del structures through experimental structural biology, additional detailed kinetics studies, and receptor signaling/crosstalk studies will be an important next step in ongoing efforts to design new treatment strategies for patients with *EGFR*-mutant NSCLC.

## Materials and Methods

### TKI Source and Preparation.

Inhibitors were purchased from Selleck Chemicals.

### Cell Culture.

Ba/F3 cells (DSMZ), PC9 (American Type Culture Collection [ATCC]), SH450 (ATCC), and HCC4006 (ATCC) were cultured in RPMI 1640 with L-glutamine (Mediatech) supplemented with 10% heat-inactivated fetal bovine serum (Thermo Fisher Scientific), penicillin (100 U/mL; Thermo Fisher Scientific), streptomycin (100 μg/mL; Thermo Fisher Scientific), and IL-3 (1 ng/mL; Thermo Fisher Scientific) until retroviral transduction and subsequent IL-3 withdrawal. Cells were grown in a humidified incubator with 5% CO_2_ supply at 37 °C. *Mycoplasma* contamination was evaluated routinely during cell culture using a VenorGeM *Mycoplasma* Detection Kit (Sigma-Aldrich).

### Generation of EGFR-Expression Constructs and Generation of Ba/F3 Cell Lines.

pBabe plasmids with EGFR ex19del mutation-encoding complementary DNAs (EGFR E746_A750, EGFR E746_S752 > V, and EGFR L747_A750 > P) and EGFR WT were purchased from Addgene. The empty page–puro retroviral vector or pabebe-EGFR mutants were transfected, along with the envelope plasmid pCMV-VSV-G (Cell Biolabs), into Plat-GP packaging cells (Cell Biolabs). Forty-eight hours after transfection, viral media were collected, and the debris was removed by centrifugation. For each separate transduction, 1 × 10^6^ Ba/F3 cells were resuspended in the viral media and supplemented with 10 µg/mL polybrene (Santa Cruz Biotechnology). Transduced cells were selected using 2 µg/mL puromycin (Invitrogen). EGFR construct expressions were checked before experiments, and only stable polyclonal populations were used.

### Quantitative Assessment of Cell Proliferation during IL-3 Withdrawal.

Ba/F3 cells that had been transduced with EGFR-expressing constructs, selected with 2 μg/mL puromycin, and growing in media containing 1 ng/mL IL-3 were washed twice with warm phosphate-buffered saline (PBS) to remove IL-3. Cells were resuspended in media without IL-3 and seeded in 96-well imaging plates at a density of 3,000 cells/well. Cells were periodically scanned in IncuCyte ZOOM every 6 h using Incucyte Nuclight Rapid Red Dye for nuclear labeling. Cell doubling values were calculated using the cell counts at each time point divided by the cell counts at the start time point.

### Immunoblot and Antibodies.

Antibody EGFR (#2232), pEGFR Y1068, pEGFR Y992, pEGFR Y1184, and horseradish peroxidase–conjugated anti-rabbit (#7074) were all purchased from Cell Signaling Technology, and the actin antibody (A2066) was purchased from Sigma-Aldrich. For immunoblotting, cells were harvested before or after ligand or drug treatment, washed using PBS, and lysed with RIPA buffer [50 mmol/L Tris HCl (pH 8.0), 150 mmol/L sodium chloride, 5 mmol/L magnesium chloride, 1% Triton X-100, 0.5% sodium deoxycholate, 0.1% sodium dodecyl sulfate, 40 mmol/L sodium fluoride, 1 mmol/L sodium orthovanadate, and complete protease inhibitors (Roche Diagnostics)]. For signal detection, Western Lightning Enhanced chemiluminescence reagent (PerkinElmer) was used. Phosphorylated bands were quantified using ImageJ.

### Viability Assays.

Experiments were conducted in the Vanderbilt High-Throughput Screening Facility. Cells were seeded at ∼800 cells per well in 384-well plates using Multidrop Combi Reagent Dispenser (Thermo Fisher Scientific). Media containing different drug concentrations were prepared using a column-wise serial 3X dilution in 384-well plates using a Bravo Liquid Handling System (Agilent) and were added to the cells. Cell viabilities were obtained using the CellTiter-Blue Cell Viability Assay (Promega).

### Statistical Analysis.

All experiments were performed at least three times, and the differences were determined by ordinary one-way ANOVA using GraphPad Prism 9.2.0. Difference was considered significant when *P* < 0.05.

### Enzymatic Analysis.

EGFR WT (#E10-112G, lot J3837-8), E746_A750 (#E10-122JG, lot O3886-10), E746_S752 > V (lot T4348-4), L747_A750 > P (#E10-12MG, lot G1200-3), and L747_E749 (#E10-12LG, lot G1344-5) were purchased from SignalChem. The Promega ADP-Glo kinase assay kit was used to quantify the amount of adenosine 5′-diphosphate (ADP) produced by each EGFR variant in 1XBFA buffer and in the presence or absence of erlotinib at varying concentrations. Poly(4:1 Glu, Tyr) at a concentration of 0.2 µM was used as the peptide substrate. Reactions were performed at room temperature for 40 min each at varying ATP concentrations: 3.125, 6.25, 12.5, 25, 100, and 500 µM. Reactions were performed on 384-well plates with each ATP concentration performed in duplicate. Following incubation for 40 min, the Promega ADP-Glo reagent was utilized to quench the enzymatic reaction and remove residual ATP. The kinase detection agent provided with the assay kit was subsequently used to convert product ADP back into ATP and measure luminescence from the ATP-powered luciferase/luciferin reaction. ATP *K*_m_ and erlotinib *K*_i_ were fit according to a mixed model of inhibition using GraphPad Prism 9.3.1. Assays were performed in collaboration with SignalChem.

### PIE-FCCS.

FCCS data were taken on a customized microscope system to introduce PIE and time-correlated single photon counting as shown in previous works (33, 41). A detailed description of the PIE-FCCS protocol is available in the Supporting Information.

### Computational Modeling.

Structural modeling of proteins was carried out using the Rosetta v.3.12 package (56, 57). MD simulations were performed with Amber18 utilizing the Amber ff14SB and GAFF2 forcefields for proteins and ligands, respectively (51, 58). We estimated protein-ligand–binding free energies using the MM-PBSA.py package in AmberTools18 (59). rmsd, atom-atom distances, and dihedrals angles were obtained using CPPTRAJ in AmberTools18. The initial structure of osimertinib was taken from Protein Databank (PDB) ID 4ZAU (48). The initial structure of neratinib was obtained from PDB ID 3W2Q (60). The structures were geometry optimized using Gaussian 09 revision D.01 at B3LYP/6–31G(d) level of theory and the electrostatic potential of the optimized structures computed with HF/6–31G(d) in the gas phase. Atomic partial charges were fit with the restrained electrostatic potential algorithm in AmberTools18. ATP parameters were developed previously (61), and coordinates were initialized from PDB ID 2ITX. For protein–ligand complexes of variants with osimertinib, neratinib, or ATP, we utilized the above PDB structures for ligand placement. Detailed modeling protocols are available in the Supporting Information.

## Supplementary Material

Supplementary File

Supplementary File

## Data Availability

Computational structural models for EGFR ex19del active state KDs, compressed MD simulation trajectories, and sample scripts are available on Zenodo under the DOI: 10.5281/zenodo.6604137 (62). Please contact the corresponding authors with additional questions.
