# Supplementary material for: Allele-specific activation, enzyme kinetics, and inhibitor sensitivities of EGFR exon 19 deletion mutations in lung cancer
Source: Proc Natl Acad Sci U S A. 2022 Jul 22;119(30):e2206588119. doi: 10.1073/pnas.2206588119 (PMC9335329; doi:10.1073/pnas.2206588119)
Supplement: Supplementary File [file pnas.2206588119.sapp.pdf]

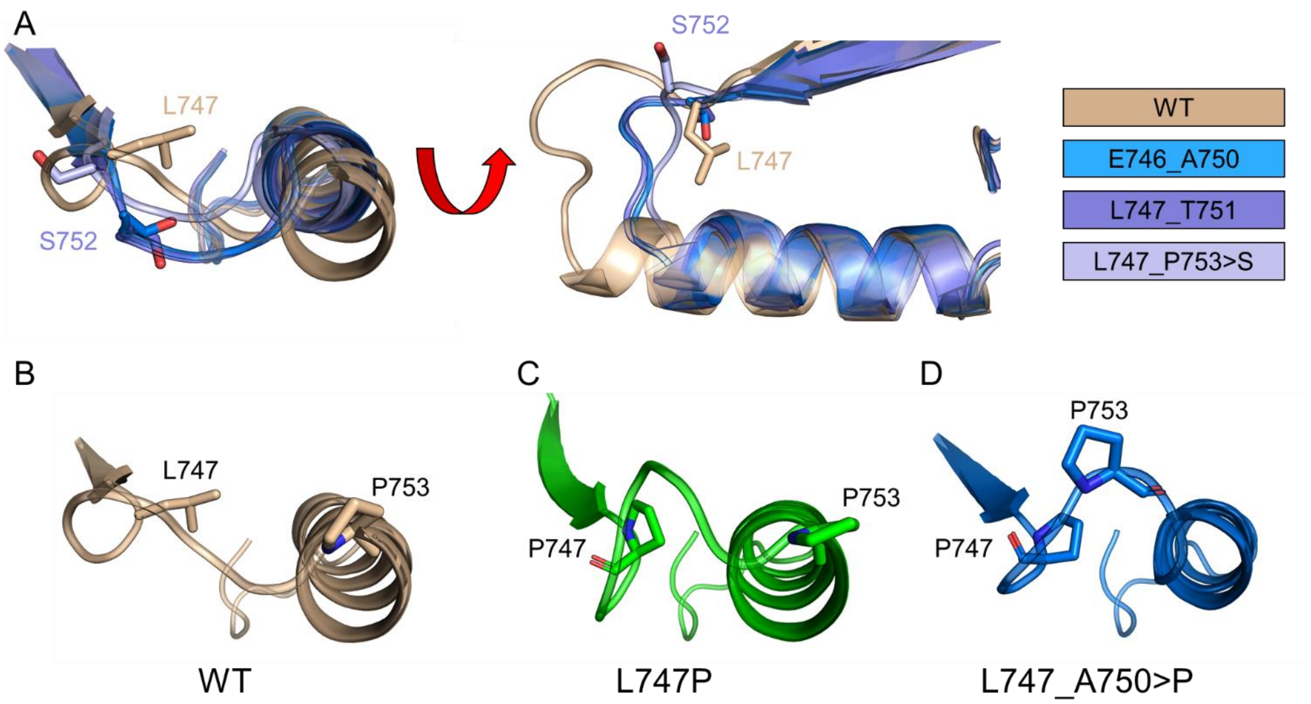

Figure S1. Structural comparison of modeled ex19del β3αC motifs. (A) Superimposition of the β3αC region of the most common ex19del variants with WT. Rendering of the β3αC loop in **(B)** WT, **(C)** L747P, and **(D)** L747_A750>P. L747P and L747_A750>P both form a tight turn in the β3αC loop. The L747_A750>P tight turn contains a proline in the second position and fewer residues on the N-terminus of the αC-helix.

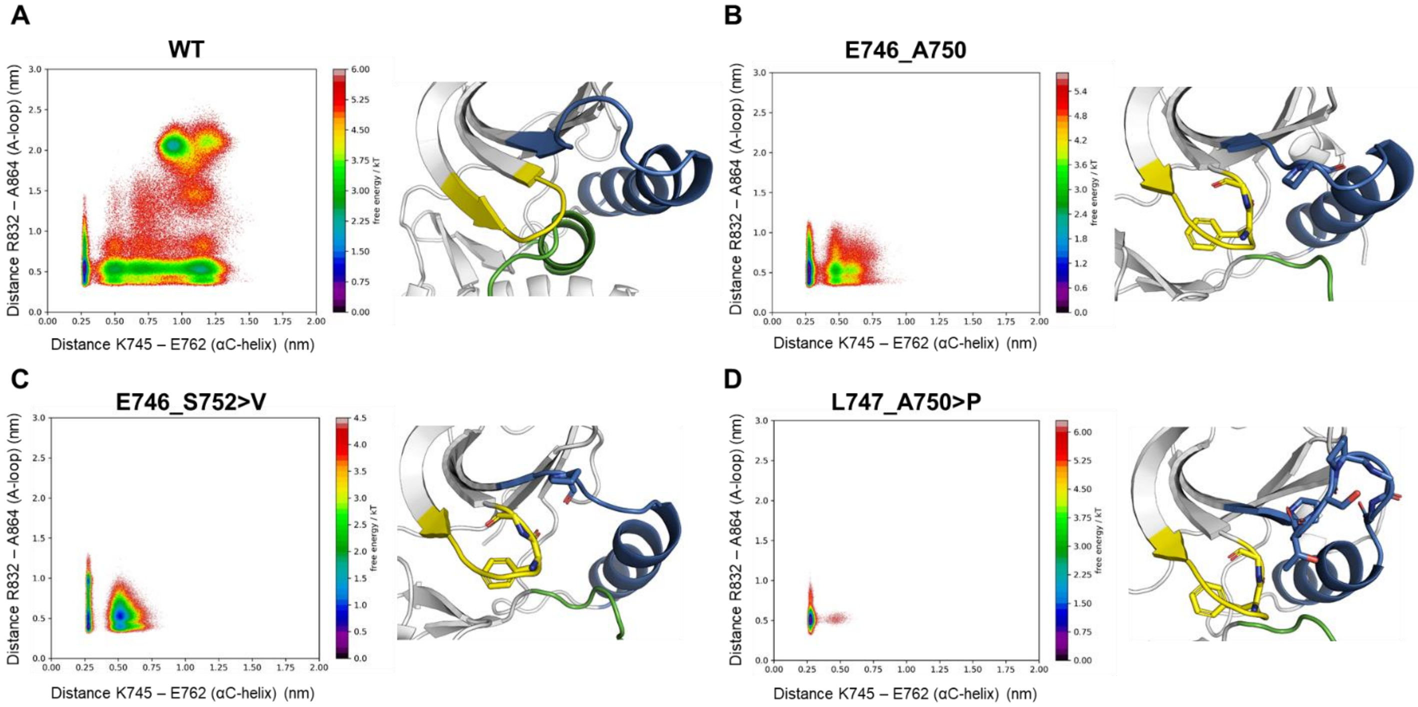

Figure S2. Conventional MD simulations of several ex19del variants starting from the active state. Boltzmann-weighted probability distributions of **(A)** WT, **(B)** E746_A750, **(C)** E746_S752>V, and **(D)** L747_A750>P conformational changes in conventional MD simulations. All simulations were started from the active state. Three independent simulations for each system were run for 4.0 us each. The inward/outward motion of the activation loop is depicted on the y-axis (larger numbers indicate more inward), and the inward/outward motion of the αC-helix is depicted on the x-axis (larger numbers indicate more outward). Snapshots are from the end of one of the three independent simulations. WT transitioned to the Src-like inactive state in one of the three simulations. The glycine-rich loop is colored yellow, the β3αC-loop and αC-helix are blue, and the activation loop is green.

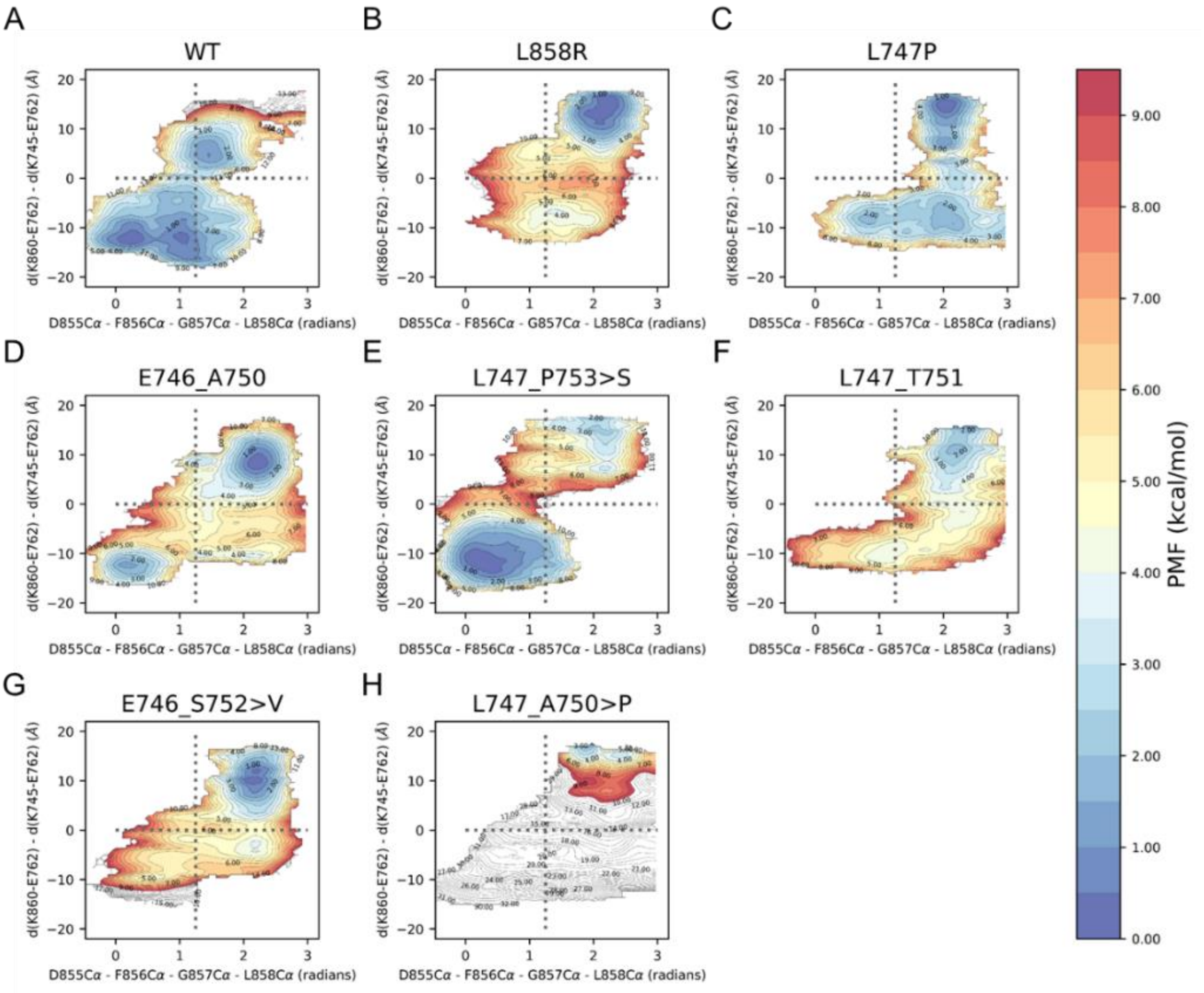

Figure S3. Conformational free energy landscapes of EGFR variants from umbrella sampling MD simulations. Collective variables describe the active and inactive states as the pseudo-dihedral angle formed by the alpha carbon atoms of residues D855, F856, G857, and L858 (x-axis) as well as the difference in distance between the capping sidechain atoms of E762 and K745 (d1) and E762 and K860 (d2) (y-axis). Conformational free energies are shown for **(A)** WT, **(B)** L858R, **(C)** L747P, **(D)** E746_A750, **(E)** L747_P753>S, **(F)** L747_T751, **(G)** E746_S753>V, and **(H)** L747_A750>P. Plots are contoured at 0.5 kcal/mol and colored within the range 0 (blue) and 9.5 (red) kcal/mol. Contours above 9.5 kcal/mol are colored white.

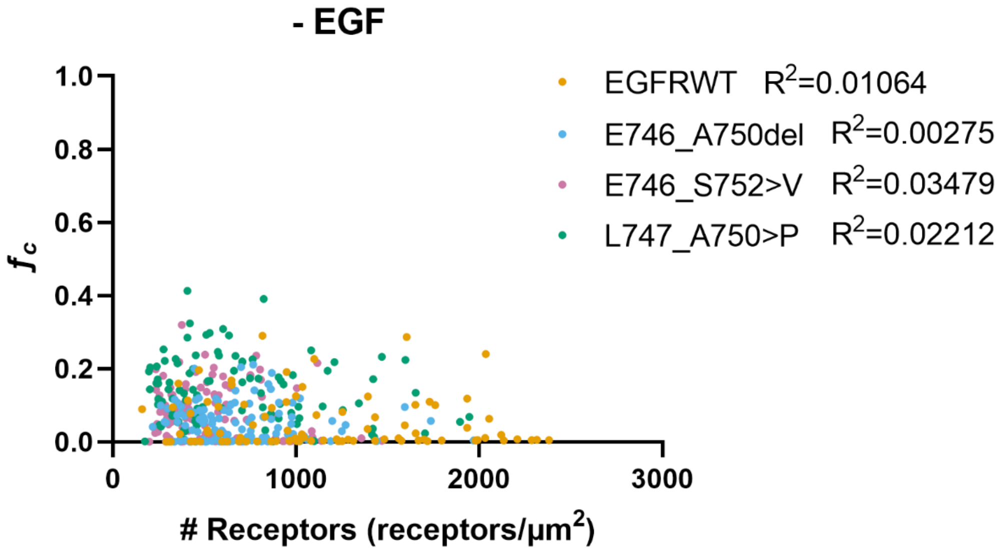

Figure S4. Single-cell protein expression levels for EGFR variants. The PIE-FCCS cross-correlation values for EGFR WT (yellow), E746_A750 (cyan), E746_S752>V (pink), and L747_A750>P (green) reported in Figure 3 are plotted here against their respective single-cell expression levels. These measurements were taken in the absence of EGF stimulation.

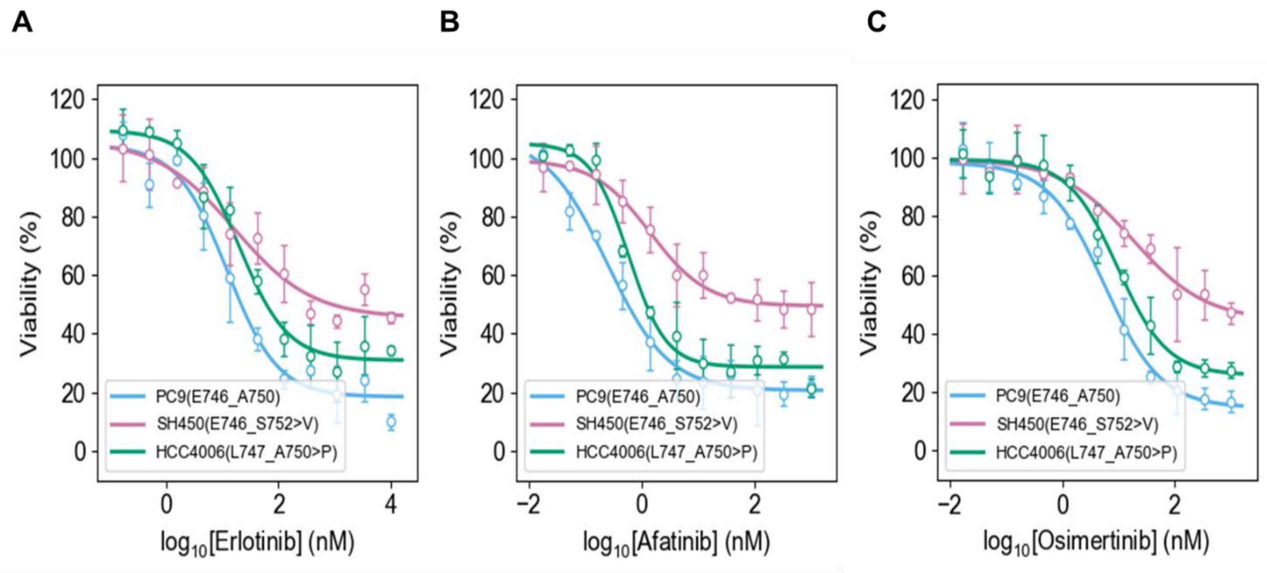

Figure S5. EGFR ex19del variants display differential responses to multiple distinct EGFR TKIs when expressed in isogenic cell lines. Cell viability assays performed in lung adenocarcinoma cells stably expressing E746_A750 (PC9; blue), E746_S752>V (SH450; pink), or L747_A750>P (HCC4006; green) with **(A)** erlotinib, **(B)** afatinib, or **(C)** osimertinib. Three biological replicates performed for each mutant/TKI combination. Representative plots displayed.

A

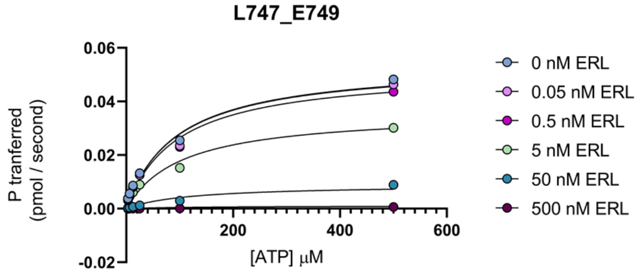

B

EGFR	ATP Km (µM)	ATP Vmax (pmol/s)	ATP Vmax/Km	ERL Ki (nM)
L747_E749	94.88 (73.75 – 116.0)	5.45E-02	5.74E-04	7.72 (0.0 – 15.67)

Figure S6. L747_E749 is kinetically more similar to E746_A750 than L747_A750>P. Michaelis-Menten steady state kinetics of EGFR **(A)** L747_E749 at varying concentrations of the ATP-competitive non-covalent tyrosine kinase inhibitor erlotinib (ERL) and substrate ATP at 0.2 µg/µL of peptide substrate Poly (4:1 Glu, Tyr) as determined by the ADP-Glo assay. Rate is expressed as phosphate transferred in pmol/second. **(E)** Enzyme kinetic parameters and erlotinib binding affinity for L747_E749. Data from **(A)** were fit with least-squares to a mixed model of inhibition in GraphPad Prism 9.3.1 and are reported as best-fit values. The value ranges in parentheses indicate 95% confidence intervals.

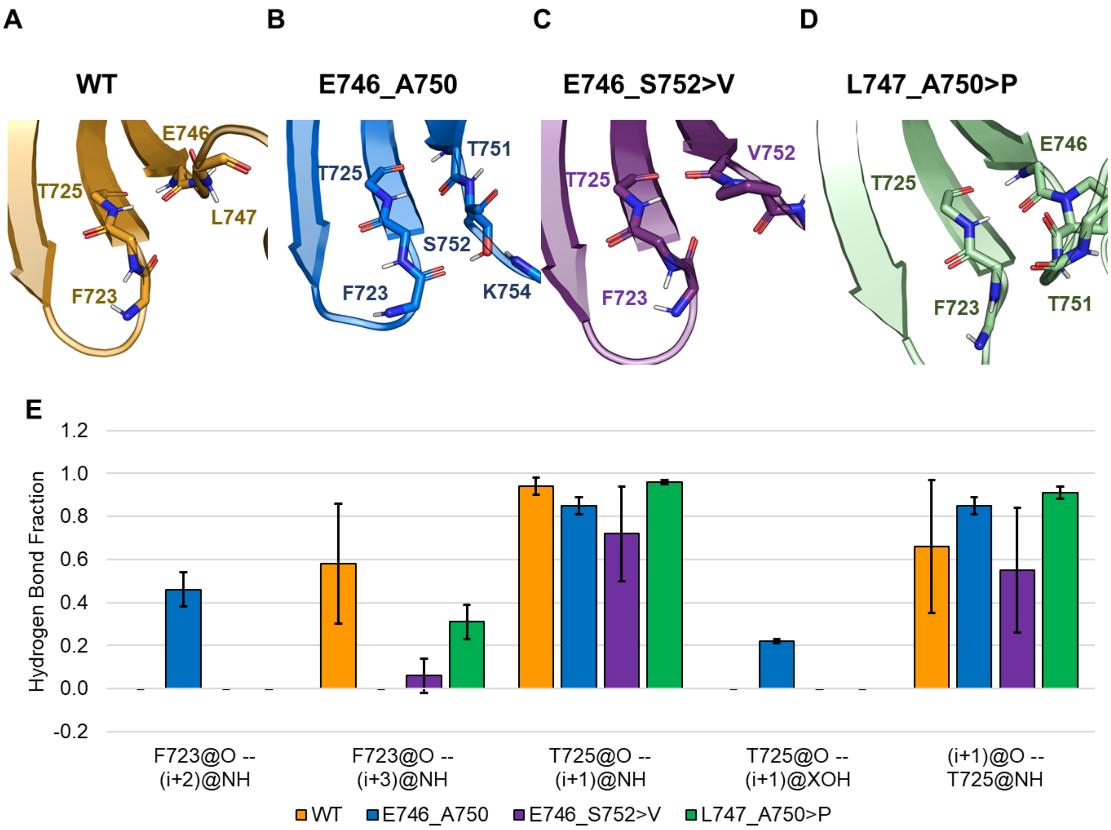

Figure S7. Conventional MD simulations demonstrate ex19del β3αC hydrogen bond networks. Apo-state conventional MD simulation snapshots of β3αC hydrogen bond networks in **(A)** WT, **(B)** E746_A750, **(C)** E746_S752>V, and **(D)** L747_A750>P. **(E)** Quantification of hydrogen bond stability of select β3αC hydrogen bonds at the interface. Hydrogen bonds are defined by donor/acceptor heavy atom distances of ≤ 3.5 and angles between 135 and 180 degrees. Quantifications are based on three independent trials of 4.0 us apo-state simulations of each system starting from the active state.

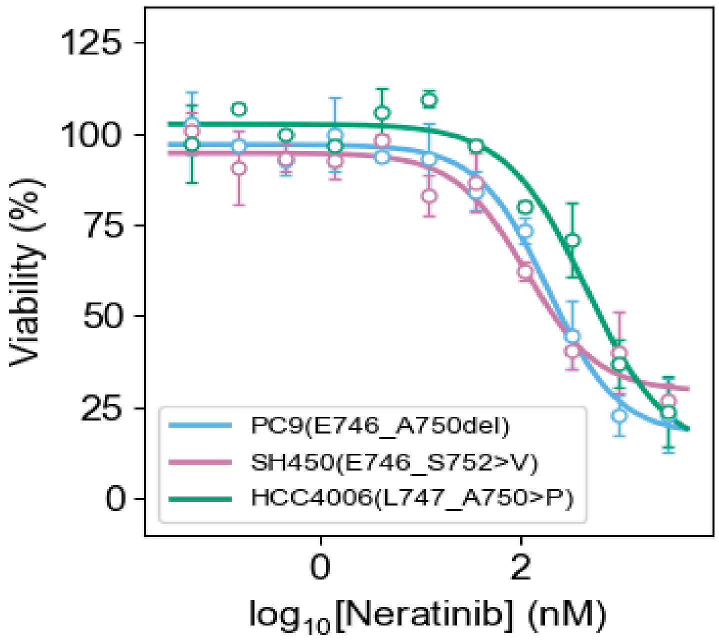

Figure S8. Lung adenocarcinoma cell lines expressing different EGFR ex19del variants are similarly inhibited by neratinib. Cell viability assays performed in lung adenocarcinoma cell lines stably expressing E746_A750 (PC9), E746_S752>V (SH450), or L747_A750>P (HCC4006) with neratinib.

Table S1. Exon 19 deletion variants identified in AACR GENIE database.

Mutation	# Cases	Frequency (%)
p.E746_A750del	857	65.07
p.L747_P753delinsS	101	7.67
p.L747_T751del	71	5.39
p.E746_S752delinsV	54	4.10
p.L747_A750delinsP	51	3.87
p.L747_E749del	24	1.82
p.S752_I759del	18	1.37
p.E746_T751delinsA	17	1.29
p.L747_S752del	14	1.06
p.L747_T751delinsP	10	0.76
p.E746_S752delinsA	9	0.68
p.E746_T751delinsI	7	0.53
p.E746_P753delinsVS	6	0.46
p.E746_T751delinsVP	5	0.38
p.T751_I759delinsN	5	0.38
p.E746_T751delinsIP	4	0.30
p.T751_E758del	4	0.30
p.E746_A750delinsAP	3	0.23
p.L747_A750del	3	0.23
p.L747_A750delinsS	3	0.23
p.E746_A750delinsP	2	0.15
p.E746_A750delinsQP	2	0.15
p.E746_E749del	2	0.15
p.E746_P753delinsLS	2	0.15
p.E746_T751del	2	0.15
p.E746_T751delinsV	2	0.15
p.E746_T751delinsVA	2	0.15
p.L747_A755delinsAT	2	0.15
p.L747_S752delinsQ	2	0.15
p.L747_T751delinsQ	2	0.15
p.T751del	2	0.15
p.A750_I759delinsPT	1	0.08
p.A750del	1	0.08
p.E746_A750delinsD	1	0.08
p.E746_A750delinsIP	1	0.08

p.E746_A750delinsRP	1	0.08
p.E746_E749delinsD	1	0.08
p.E746_E749delinsQ	1	0.08
p.E746_P753delinsIS	1	0.08
p.E746_S752delinsD	1	0.08
p.E746_S752delinsI	1	0.08
p.E746_T751delinsAPS	1	0.08
p.E746_T751delinsVS	1	0.08
p.K739_I744delinsN	1	0.08
p.L747_A750delinsEP	1	0.08
p.L747_A755delinsAN	1	0.08
p.L747_A755delinsSES	1	0.08
p.L747_A755delinsSKD	1	0.08
p.L747_A755delinsSMS	1	0.08
p.L747_E749delinsQ	1	0.08
p.L747_P753delinsQPS	1	0.08
p.L747_S752delinsQH	1	0.08
p.L747_S752delinsQP	1	0.08
p.L747_T751delinsN	1	0.08
p.L747_T751delinsPA	1	0.08
p.L747_T751delinsPR	1	0.08
p.L747_T751delinsS	1	0.08
p.P753_I759del	1	0.08
p.T751_A755del	1	0.08
p.T751_I759delinsS	1	0.08
Total	1317	N/A

Movie S1. Conventional molecular dynamics (cMD) simulation of active-to-inactive state conformational transition of EGFR wild-type (WT). Single 4.0 μs cMD trajectory of EGFR WT starting in the active (αC-helix in, activation loop out) state. This is one of three independent 4.0 μs EGFR WT trajectories begun from the active state and the only one that transitioned to the activation loop inward conformation. Key structural motifs colored as follows: αC-helix – purple, β3αC-linker – blue, glycine-rich loop – yellow, activation loop – green.

Pulsed Interleaved Excitation Fluorescence Cross-Correlation Spectroscopy (PIE-FCCS)

FCCS data were taken on a customized microscope system to introduce pulsed interleaved excitation (PIE) and time-correlated single photon counting (TCSPC) as shown in previous works (1, 2). A supercontinuum pulsed fiber laser (9.74 MHz repetition rate, SuperK EXW-12 NKT Photonics, Birkerød, Denmark) was split into 488 nm and 561 nm beams using filters and mirrors for the excitation of eGFP and mCherry, respectively. The 50 ns time delay for PIE was introduced by directing the split beams through two different-length optical fibers (3). The beams were overlapped and directed to the microscope. A 100X TIRF oil objective (Nikon, Tokyo, Japan) was used to focus the excitation beam and collect the fluorescence emission. NIST traceable fluorescein (50 nM; Thermo Fisher Scientific) was used for optical path alignment, and a short, fluorescent-tagged DNA was used for both alignment and as a cross-correlation control. Previously published membrane protein monomer and dimer controls (1, 2) were tested regularly for data quality control and comparison between fit parameters. The overlapped excitation beams were focused on the plasma membrane of COS7 cells transfected with mCherry- and GFP-labeled EGFR. A z-axis scan was done to ensure that the laser beam was focused on the flat, peripheral membrane area. One 60-second data acquisition was taken per cell. The emitted fluorescence was collimated, separated, and filtered before being focused onto two single-photon avalanche diodes (Micro Photon Devices, Bolzano, Italy). A TCSPC module (Picoharp 300, PicoQuant, Berlin, Germany) recorded the photon counts for each channel. For analysis, the time-tagged photon counts were divided into 10-second segments that were then binned and gated for removal of spectral cross-talk. Auto- and cross-correlation curves corresponding to each species were calculated with a custom MATLAB script. The correlation functions from each acquisition were averaged and then fitted to a single component, 2D diffusion model as shown previously (1, 3, 4). From the fits we used the average dwell time (τ_D) to calculate the effective diffusion coefficient, $D_{eff} = \omega_o^2/4\tau_D$. The amplitude of the curves was used to calculate the local concentration of the diffusing receptors in the detection area. From the ratio of the auto and cross-correlation amplitudes, we calculated a cross-correlation value (f_c) for each cell, with which we assess the degree of oligomerization. Based on a previous calibration using a live cell control system, the expected f_c value for a monomer-dimer equilibrium is 0.10 to 0.15 (3). Higher f_c values indicate higher order oligomerization (3).

EGFR ex19del structural modeling

We first built structural models of the 60 ex19del variants identified in AACR GENIE with RosettaCM using the REF2015 score function(5). As templates, we selected the active state EGFR WT structures from PDB IDs 2ITX and 2GS6. We also used the active state model of L858R from PDB ID 4I20. We also included as templates the MD equilibrated structural models of E746_A750 and E746_S752>V we made for our prior study (6). We generated 5,000 RosettaCM models for each variant. The best scoring variant from each was simulated with GaMD for 1.0 us (60.0 us total). GaMD simulation trajectories were clustered with DBSCAN in CPPTRAJ based on β3αC loop RMSD. Each variant was subsequently remodeled with RosettaCM to generate 10,000 more models using the DBSCAN cluster centers as additional templates alongside the prior templates. The best scoring model in round two is the final model. Active state L747P was modeled as a point mutation using the Rosetta PackRotamersMover and FastRelax mover starting from EGFR WT in PDB ID 2ITX. We performed a 1.0 us GaMD simulation on the resulting L747P structure, followed by DBSCAN clustering with CPPTRAJ as above. A representative structure from each cluster was relaxed in Rosetta with progressively ramped-down constraints to the starting coordinates to produce 50 models for each cluster. The best scoring model was carried forward for additional simulations. Inactive state structural models of E746_A750, E746_S752>V, L747_A750>P, L747_T751, L747_P753>S, and L747P were modeled with RosettaCM using the inactive state symmetric dimer EGFR WT in PDB ID 3GT8 as a template.

Conventional MD (cMD) simulations

Each structure was solvated in a rectangular TIP3P box (12 Å buffer) neutralized with monovalent Cl^- and Na^+ ions (7). Minimization proceeded in three stages: solvent minimization with constraints on solute atoms, solute minimization with constraints on solvent, and subsequently full system minimization without constraints. Each of these stages consisted of 1,000 steps of steepest gradient descent followed by 4,000 steps of conjugate gradient descent. The system was heated in the canonical (NVT) ensemble to 100 K over 100 ps. The system was then heated in the isothermal-isobaric (NPT) ensemble at 1 bar from 100 K to physiologic 310 K over 400 ps. Equilibration was performed in NPT ensemble at 310K for an additional 1000 ps. NPT simulations utilized a Monte Carlo barostat. The temperature was controlled using Langevin dynamics with a collision frequency of 2.0 ps^{-1}. A unique random seed was used for each simulation. SHAKE was implemented to constrain bonds involving hydrogen atoms. Periodic boundary conditions were applied

and the particle mesh Ewald (PME) algorithm was adopted for long-range electrostatics with a switching distance of 10 Å. Hydrogen mass repartitioning was employed on solute atoms to allow an integration time step of 4 fs.

Gaussian Accelerated MD (GaMD) simulations

Gaussian accelerated MD (GaMD) is an enhanced sampling method that adds a boost potential to the potential energy surface to accelerate transitions between low-energy states (8, 9). The dual boost potential scheme was applied to the system in order to enhance conformational sampling (9). Systems were equilibrated for 50 ns in cMD. Subsequently, potential statistics for GaMD acceleration were computed from a 10 ns cMD simulation. After addition of the GaMD boost potential, simulations were equilibrated for an additional 50 ns before production. All GaMD simulations were performed in NVT ensemble with a Langevin thermostat and collision frequency of 5.0 ps^{-1}. The upper limit of the boost potential standard deviation was set to 6.0 kcal/mol.

Umbrella sampling and conformational free energy landscapes

Conformational free energy landscapes (FEL) of EGFR WT and ex91del mutants were obtained with constant velocity steered MD (SMD) coupled with Umbrella sampling (US) simulations. The weighted histogram analysis method (WHAM) as implemented by Alan Grossfield (10) was used to perform final statistical reweighting of the US simulations. SMD simulations of 100 ns were performed with a harmonic bias potential and spring constant of 1000 kcal/mol/$Å^2$. SMD simulations were performed from the active to the inactive state and vice versa using Cα RMSD to the reference coordinates as the collective variable. A minimum of 250 windows were selected from each forward and backward simulation with which to seed US simulations. Therefore, a total of at least 500 windows per system were used to ensure overlap. A 2D harmonic restraining potential was applied to two CVs for the US simulations. CV1 (y-axis) was defined as the difference in the distance between K860(NZ) – E762(OE1, OE2) and K745(NZ) – E762(OE1, OE2). CV2 (x-axis) was defined as the dihedral angle formed by the Cα atoms of the following residues: D855, F856, G857, and L858. A 2.0 kcal/mol/$Å^2$ spring constant was used for CV1, and a 10.0 kcal/mol/rad^2 spring constant was used for CV2. At each umbrella center a 5 ns simulation was performed. The first 1 ns was used for equilibration, and the following 4 ns were used for analysis in WHAM. Lowest free energy pathway (LFEP) analysis completed with the LFEP package freely available from the Moradi Laboratory at the University of Arkansas.

Binding free energy calculations

The estimated binding free energies between EGFR and TKI (osimertinib or neratinib) was computed with the molecular mechanics Poisson-Boltzmann surface area (MM-PBSA) method using the MMPBSA.py program in AmberTools18 (11). From the equilibrated trajectories, we randomly resampled 1,000 structures to use for binding free energy calculations. For the MM-PBSA calculations, the internal and external dielectric constants were set to 2.0 and 80.0, respectively. The nonpolar component of the solvation free energy was decomposed into separate cavity formation and dispersion terms (INP=2) with default cavity offset and surface tension values. Ionic strength was set to 150 mM. Atomic radii were taken from pre-computed Amber values (RADIOPT=1).

References

1. N. F. Endres *et al.*, Conformational coupling across the plasma membrane in activation of the EGF receptor. *Cell* **152**, 543-556 (2013).
2. Y. Huang *et al.*, Molecular basis for multimerization in the activation of the epidermal growth factor receptor. *Elife* **5** (2016).
3. W. D. Comar, S. M. Schubert, B. Jastrzebska, K. Palczewski, A. W. Smith, Time-resolved fluorescence spectroscopy measures clustering and mobility of a G protein-coupled receptor opsin in live cell membranes. *J. Am. Chem. Soc.* **136**, 8342-8349 (2014).
4. M. J. Kaliszewski *et al.*, Quantifying membrane protein oligomerization with fluorescence cross-correlation spectroscopy. *Methods* **140-141**, 40-51 (2018).
5. R. F. Alford *et al.*, The Rosetta All-Atom Energy Function for Macromolecular Modeling and Design. *J. Chem. Theory Comput.* **13**, 3031-3048 (2017).
6. B. P. Brown *et al.*, On-target Resistance to the Mutant-Selective EGFR Inhibitor Osimertinib Can Develop in an Allele-Specific Manner Dependent on the Original EGFR-Activating Mutation. *Clinical cancer research : an official journal of the American Association for Cancer Research* **25**, 3341-3351 (2019).
7. S. Yoshikawa *et al.*, Structural basis for the altered drug sensitivities of non-small cell lung cancer-associated mutants of human epidermal growth factor receptor. *Oncogene* **32**, 27-38 (2013).
8. A. B. Hanker *et al.*, Co-occurring gain-of-function mutations in HER2 and HER3 modulate HER2/HER3 activation, oncogenesis, and HER2 inhibitor sensitivity. *Cancer Cell* https://doi.org/10.1016/j.ccell.2021.06.001 (2021).
9. Y. Miao, V. A. Feher, J. A. McCammon, Gaussian Accelerated Molecular Dynamics: Unconstrained Enhanced Sampling and Free Energy Calculation. *J. Chem. Theory Comput.* **11**, 3584-3595 (2015).
10. Y.-T. Lin, J.-Y. Shih, Not All EGFR Exon 20 Insertions Are Created Equal. *JTO Clinical and Research Reports* **1** (2020).
11. B. R. Miller *et al.*, MMPBSA.py: An Efficient Program for End-State Free Energy Calculations. *J. Chem. Theory Comput.* **8**, 3314-3321 (2012).
